# Optoelectronic Enhancement in Nanostructured h‑BN Synthesized Using Pulsed Ultrasonication

**DOI:** 10.1021/acsomega.5c12635

**Published:** 2026-03-18

**Authors:** Albin Tony, Rajib Mahato, Anagh Bhaumik

**Affiliations:** Department of Materials Engineering, 242275 Indian Institute of Technology Gandhinagar, Gandhinagar 382055, India

## Abstract

This work describes the synthesis of hexagonal boron nitride nanoparticles (h-BN NPs) and their use as a surface-modifying layer to improve the optoelectronic responsivity in the UV region. We have shown by optoelectronic measurements that nano structuring h-BN promotes effective photon interaction, improving the enhancement factor. The synthesized h-BN NPs using the pulsed mode (frequency of 40 kHz) of ultrasonication yield an average size ∼40 nm, as evident from the field emission scanning electron microscopy (FESEM) and high-resolution transmission electron microscopy (HRTEM) analysis. Devices coated with h-BN NPs synthesized by pulsed ultrasonication exhibit an enhancement ratio (*R*_OFF_/*R*_ON_) of ∼35% higher than those of commercially available light-dependent resistors (LDRs). Detailed structure–property analysis employing Raman spectroscopy, X-ray diffraction (XRD), X-ray photoelectron spectroscopy (XPS), dynamic light scattering, FESEM, and HRTEM supports the role of size, chemical composition, and bonding characteristics of h-BN NPs in improving UV sensitivity. XPS data reveal increased defect concentration in the pulsed and non-pulsed synthesized h-BN NPs due to reduced B–N XPS intensity. Furthermore, O atoms have a greater affinity toward N atoms in pulsed h-BN as compared to non-pulsed h-BN where O atoms bond to B atoms. Raman spectroscopy and XRD also reveal the characteristic peaks of the synthesized h-BN nanostructures. The measured response and decay times of the pulsed h-BN NP-coated devices are ∼4 and ∼121 ms, respectively, which is comparable to commercial LDRs. A detailed comparative analysis of optoelectronic and structural properties of the pulsed h-BN NPs with non-pulsed h-BN NPs and commercially available h-BN was also performed. Variability and reliability analysis of the enhancement ratio in the pulsed h-BN NP-coated LDRs indicate a standard deviation of 0.05 and no appreciable change over a time of more than 15 min, respectively. To conclude, we have developed a unique synthesis technique of nanostructured h-BN for improved optoelectronic performance in the UV region with potential relevance to semiconductor and quantum applications.

## Graphical abstract

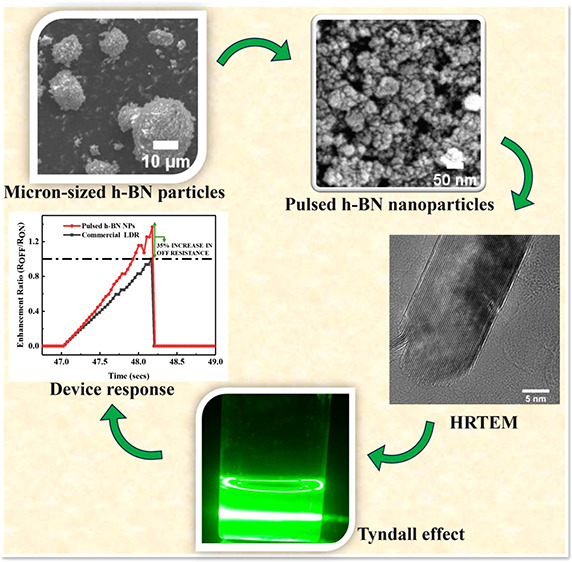

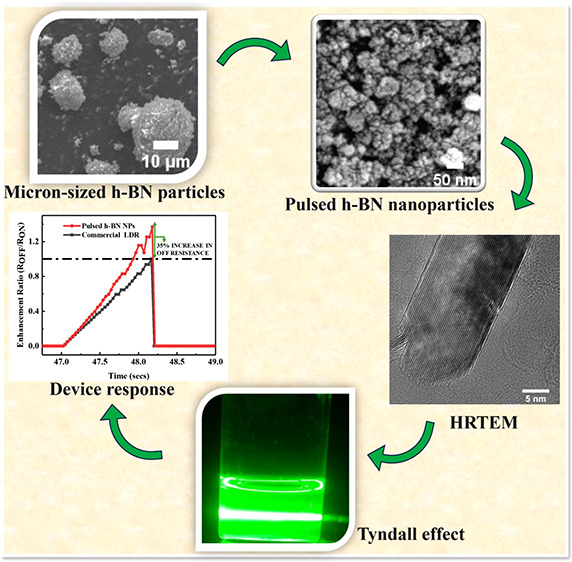

## Introduction

Ultraviolet photodetectors play a significant role in a wide range of technologies, like environmental monitoring,^1^ space exploration,^2^ semiconductor manufacturing,^1^ water purification systems,^3^ biochemical sensing,^2^ flame detection, secure optical communication, and UV-based threat detection. The most used photodetectors are silicon-based,^4^ but they have poor UV responsivity due to shallow absorption depths, high surface recombination, elevated dark current, and long-term degradation under intense UV exposure and high temperatures.^5^ They also require costly high-pass optical filters due to their narrow bandgap to detect UV. Wide-gap semiconductor materials, such as ZnO, GaN, AlGaN, TiO_2_, and SiC, are used for UV detection due to their strong UV absorption and intrinsic solar-blind characteristics.^6^ Among these materials, ZnO is widely used due to its wide band gap of ∼3.37 eV and a large exciton binding energy of 60 meV.^7^ One of the key challenges associated with ZnO nanoparticle-based photodetectors is their prolonged rise and decay times, arising from the slow adsorption and desorption of oxygen species at the ZnO surface.^8^ III–V compound semiconductors like AlN, GaN, and BN possess ultrawide band gaps and are recognized as highly promising materials for ultraviolet detectors.^9^ They have excellent sensitivity and superior resistance to high-energy radiation. Boron nitride (BN) is regarded as one of the most promising inorganic materials of this century, with a broad spectrum of applications ranging from aviation to healthcare. High thermal stability, low coefficient of friction, and low chemical reactivity make this one of the inorganic materials with diverse applications.^10^ Literature primarily discusses four types of BN, one is in amorphous form (a-BN), and the other three types of BN are in crystalline form; they are hexagonal boron nitride (h-BN), cubic boron nitride (c-BN), and wurtzite boron nitride (w-BN).^11^ H-BN, being a ceramic material, was traditionally used in thermal applications like heat shielding, high-temperature lubrication and as a filler material in composite materials for heat dissipation.^12^ Optoelectronics-based research on graphene has significantly advanced the study of other monolayer materials that exhibit weak van der Waals forces.^13^ H-BN has emerged as a 2D material with numerous applications in nanoelectronics. Its large bandgap, stable unreactive surface, and moderate dielectric constants are properties that make it a desired material for optoelectronic and photonics applications.^14^ It is also used as a neutron-absorbing material due to the presence of boron in it.^15^ The defects at the atomic scale of h-BN could also be used as a source for a single-photon emitter,^16^ which is the prime component of quantum communication. Compared with bulk h-BN, the 2d layered h-BN structures possess good properties in terms of photon emission due to less interaction with the bulk material, and it is easy to integrate them into the on-chip photonics for quantum computing applications.^17^ Negatively charged boron vacancies in h-BN have great potential in quantum-sensing applications. Thus, the two-dimensional (2D) hexagonal boron nitride has become a significant material for electronic applications and ultraviolet detection.^18^ High-quality h-BN exhibits a low defect density and minimal persistent photoconductivity, which contributes to stable and reproducible performance over an extended period.^19^ h-BN-based photodetectors can show quicker response and recovery times. This happens because they lack the strong surface oxygen adsorption effects found in metal-oxide semiconductors. The use of high-quality crystals or nanostructures further enhances this performance.^20^ The wide bandgap, excellent chemical stability, and easy integration with thin-film devices of h-BN are some of the main reasons for its increasing interest in ultraviolet photodetectors, especially in the DUV range. Early work illustrated vertical MSM DUV photodetectors fabricated using laser-plasma deposition of h-BN nanosheets, proving the ability of BN nanosheets to work effectively as an active UV-sensing medium within MSM architectures.^21^ Since then, great improvement has been achieved in enhancing the major key performance metrics: for example, Veeralingam et al. present a flexible DUV photodetector based on solid-state-assisted synthesized h-BN nanosheets with record responsivity and detectivity values, pointing out that high-quality nanosheets combined with proper device engineering hold the key toward high performance.^22^ Beyond purely BN devices, h-BN has also been used in many hybrid systems as a photosensitizing or photogating layer to enhance the DUV response. One important representative work is that by Fukushima et al., where CVD-grown h-BN was used to achieve high-performance photogating for ultrahigh responsivity graphene-based DUV photodetectors.^23^ Its chemical resistance ensures nondegradation against environmental factors, and its moderate dielectric value supports its use in optoelectronic devices. H-BN also features a very high band-edge absorption coefficient (7.5 × 10̂^5^ cm^–1^).^24^ H-BN exhibits strong absorption in the DUV spectral region, making it a suitable material for UV-sensing applications. However, the high deposition temperature restricts the choice of h-BN film substrates and hinders the fabrication of h-BN photosensing devices.^25^ Due low catalytic activity and lattice mismatch of dielectric substrate, h-BN is difficult to nucleate directly, resulting in poor crystallinity of the h-BN layer for the h-BN layer synthesized by using methods like chemical vapor deposition (CVD), ion beam sputtering deposition (IBSD), and molecular beam epitaxy (MBE).^26^

Nanoparticles (NPs) and quantum dots (QDs) have been extensively employed to improve the performance of photodetectors due to their high surface-to-volume ratio, size-dependent optical absorption, and rich interfacial states, which can be utilized to enhance photocarrier generation and transport via charge transfer and trap-assisted gain processes. In UV photodetectors, ZnO QDs have been used as UV-absorbing materials in their own right, allowing for strong UV responsivity in small device sizes.^27^ Similarly, solution-processed UV photodetectors using ZnO nanoparticles in composite active layers have also shown that the integration of nanomaterials can significantly enhance UV detection capabilities by increasing absorption and providing efficient carrier separation/transport channels.^28^ More recently, oxide-QD heterostructures (e.g., ZnO-QD) have been demonstrated to further enhance the efficiency of UV photodetectors via interfacial band engineering and improved carrier dynamics.^29^

In this work, hexagonal boron nitride (h-BN) nanoparticles with sizes in a range of 30–50 nm were synthesized using a pulsed ultrasonication method. The synthesized h-BN nanoparticles were subsequently coated onto a CdS-based light-dependent resistor (LDR), and the variation in device resistance under light ON and OFF conditions was systematically investigated. The h-BN nanoparticle coating significantly enhanced the device’s ultraviolet (UV) light-sensing performance.

## Experimental Section

### Synthesis of h-BN NPs

A total of 20 mg of h-BN powder (purchased from Sigma-Aldrich, CAS no. 10043-11-5), with an average particle size of 5 μm, was measured and mixed with 10 mL of deionized (DI) water. The resulting solution was subjected to ultrasonication (Inkarp Ultrasonic Bath Model INK 180DM-6.5L, 144Watt power at 40 kHz frequency) in both normal and pulse mode for 2 h. In continuous operation mode, the ultrasonicator operated uninterruptedly for an overall period of 2 h. In pulsed operation mode, the device was programmed to provide ultrasonic irradiation with a 50% duty cycle, with 1 s of irradiation (ON) followed by 1 s of no irradiation (OFF) for 2 h. After ultrasonication, the mixture was transferred to a centrifuge tube and centrifuged at 3000 rpm for 30 min (Figure 1b). The g-force experienced by the sample was 750 G. Following centrifugation, the top layer of liquid was carefully separated from the tube and filtered through filter paper. The resultant h-BN NP solution was used for further experimentation.

**Figure 1:**
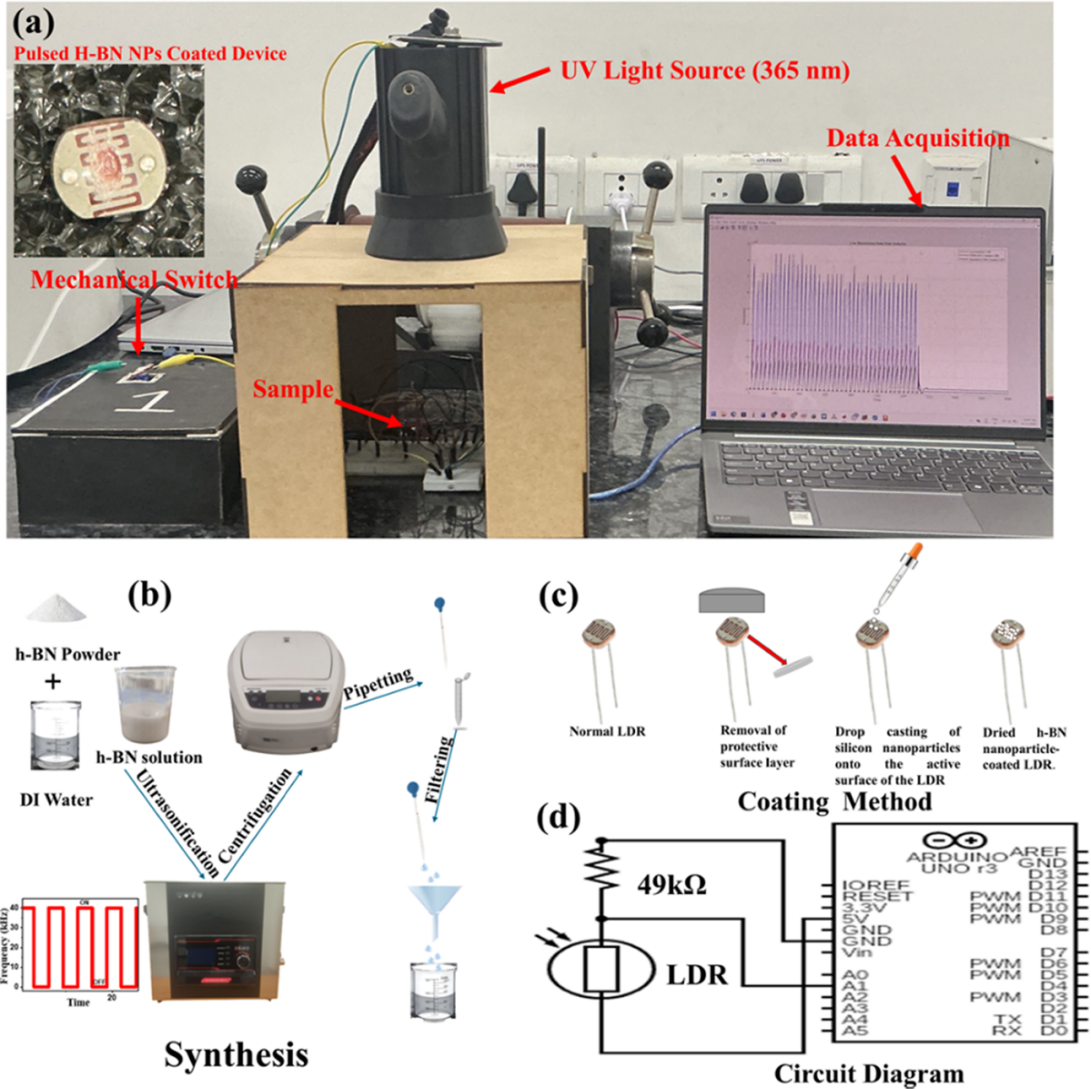
(a) Picture of the actual experimental setup used in this work; inset image shows pulsed h-BN-coated device. (b) Schematic representation of the preparation process. (c) Schematic illustration of the LDR surface modification process. (d) Electrical wiring/circuit diagram used for resistance monitoring.

### Characterizations of the h-BN NPs

The structural, morphological, and electrical properties of the h-BN NPs were analyzed by using various analytical methods. The surface texture, topography, and size distribution of h-BN NPs were investigated by using a JEOL JSM-7900F advanced analytical field emission scanning electron microscope (FESEM) at different electron-accelerating potentials ranging from 5 to 15 kV. The size distribution data were processed and plotted using ImageJ software. A Bruker AXS D8 DISCOVER powder X-ray diffraction (PXRD) system with Cu Kα radiation was used to gather crystallographic information. A Renishaw inVia spectrometer fitted with a 532 nm laser was used to record the Raman spectra. TEM images and diffraction patterns were obtained using a JEOL Themis transmission electron microscopy (TEM) with an accelerating voltage of 300 keV. X-ray photoelectron spectroscopy (XPS) was carried out using a Thermo Scientific system with an Al Kα excitation source (1486.6 eV), with all binding energies referenced to the C 1s peak at 284.6 eV with an accuracy of ±0.1 eV. The DLS experiment was done using Nano Z5 (Malvern Instruments).

### Electrical Measurement System

The synthesized h-BN NP solution is coated on the top of a commercially available CdS (cadmium sulfide)-based LDR after removing its passivation layer of the LDR (Figure 1c). A volume of **7**μL of the sample was drop-casted on the device and subsequently dried overnight without heating. The inset image of Figure 1a shows the pulsed h-BN coated device. The resistance vs time data was obtained for the sample. UV light with a wavelength of 365 ± 5 nm (Arora AUL 100 UV light source, 5000 μW/cm^2^ ± 5%) was used to illuminate the device. The experimental setup is as follows: the coated LDR was connected to an Arduino Uno microcontroller, and data were collected for resistance versus time during an ON–OFF cycle under UV light illumination (Figure 1d). The LDR was interfaced to Arduino Uno by using a voltage divide configuration (Figure 1c). The LDR was connected in series with a resistance of 49 KΩ. Also, the voltage drop across LDR was measured using the Arduino’s 10-bit ADC (0–1023 counts) referenced to 0–5 V. The LDR resistance was calculated from the measured voltage drop (divider relation), enabling direct conversion of the acquired voltage time trace into *R*–*t* data. Resistance data were acquired from an Arduino–MATLAB setup via a serial interface (4800 baud rate). Each transmission contained both a timestamp and a resistance value. A total of 5000 valid readings were taken with a sampling rate of 30 readings per second. The UV light source was ON and OFF for a specific interval of time (1 s), and data was collected for studying the *R*_OFF_/*R*_ON_ ratio for the devices. To validate the Arduino-derived resistance values, we cross-verified the resistance readings using a calibrated benchtop digital multimeter (Keysight 34450A, 51/2-digit) under identical conditions. The multimeter measurements showed close agreement with the Arduino-derived resistance values within experimental uncertainty, confirming that the Arduino–MATLAB acquisition reliably captures the transient *R*–*t* response. The resistor exhibited a lower resistance when exposed to UV light and a higher resistance value when the UV light was OFF. The photogenerated electrons were transported via the externally applied biased voltage. Similar readings were also taken for an LDR coated with standard h-BN powder. The measurement setup is shown in Figure 1a. All readings were taken under the same conditions for different devices, which helped in reducing the operator-dependent variability. The boundary conditions chosen for this experiment operation at room temperature in an ambient atmosphere and with periodic dark and light cycles are quite realistic for actual UV sensor operation.

## Results and Discussion

Figure 2a shows the Tyndall effect shown by the synthesized h-BN NPs by using green lasers. The Tyndall effect occurs when particles are smaller than the wavelength of light, leading to light scattering and making the beam path visible.^30^ Both pulsed and non-pulsed samples show the Tyndall effect, while standard particles do not show the Tyndall effect. The X-ray diffraction (XRD) pattern of the samples is shown in Figure 2b. There is a slight shift of around 0.11 ° in the peak position of the characteristic peak of h-BN. Raman spectroscopy of the particles is shown in Figure 2c that shows the characteristics E_2g_ peaks of the h-BN. Raman analysis was conducted for both pulsed and non-pulsed samples at different spot position. Raman for pulsed h-BN NPs was taken at both low and high concentrations (around 2× of the low). Here, we could see that as the concentration increases, the intensity of Raman peak also increases. Together, the results confirm that the particles that are prepared are h-BN NPs.

**Figure 2:**
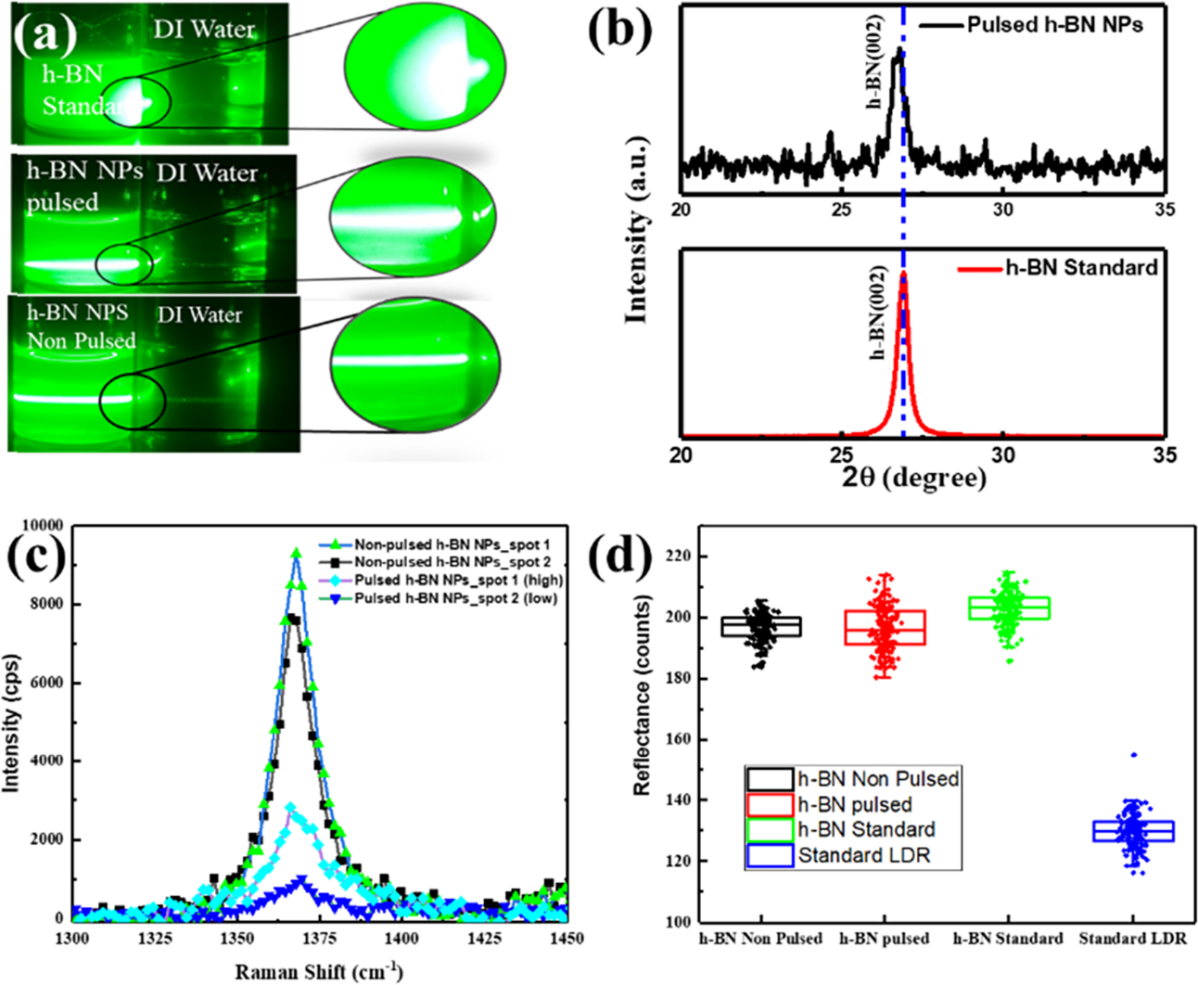
(a) Tyndall Effect (green laser) for standard h-BN powder, pulsed, and non-pulsed h-BN NPs, (b) XRD peaks of h-BN nanoparticles and standard h-BN, and (c) Raman spectroscopy of standard h-BN particles and h-BN NPs, (d) Reflectance measurement of prepared devices.

Figure 2d represents the reflectance measurements of the prepared devices. Reflectance measurements were done using an optical microscope with a 50× objective lens. The LDR samples, consisting of uncoated, standard h-BN-coated, non-pulsed h-BN-coated, and pulsed h-BN-coated variants, were imaged under the same illumination intensity, magnification, focusing, and camera exposure. The illumination of the microscope was kept constant during all measurements. The reflected intensity was determined from the images using grayscale intensity values (counts) obtained along a specified line profile on the active surface of the devices. The intensity counts are a measure of relative reflectance rather than absolute reflectance. Since all acquisition parameters were kept constant, the differences in intensity counts are a measure of relative differences in surface reflectivity among the samples. Analysis of the reflectance measurements shows that the h-BN-coated samples (standard, non-pulsed, and pulsed) have similar reflectance values over the measured distance range. It is important to note that the pulsed h-BN nanoparticle-coated device does not show a marked decrease in reflectance compared to the other h-BN coatings. The variations are small and do not indicate a large increase in optical absorption. Thus, the h-BN layer does not act as an antireflective coating on the surface of the LDR.^31,32^

Figure 3a displays the scanning electron microscope (SEM) images of the standard h-BN sample (purchased from Sigma-Aldrich, CAS no. 10043-11-5). It could be seen that there is a wide range of distribution for the size of the particles, varying from 5 to 20 μm. Figure 3b shows the size distribution of pulsed h-BN NPs. Figure 3c shows the SEM image of h-BN NPs produced by the non-pulsed ultrasonification method. Particle size analysis was performed using ImageJ and is included in the inset images of Figure 3b,c. The pulsed process yields an average particle size of ∼50 nm. Figure 3d compares the dynamic light scattering (DLS) results for commercial h-BN, nonpulsed h-BN nanoparticles, and pulsed h-BN nanoparticles. The pulsed h-BN sample exhibits a mean hydrodynamic diameter of ∼95 nm, which is larger than the average size estimated from SEM. This discrepancy is expected, as DLS measures the hydrodynamic diameter of particles in suspension and is sensitive to particle agglomeration, thereby yielding higher apparent sizes than electron microscopy. To evaluate process repeatability, DLS measurements were performed on three independently prepared pulsed h-BN NPs batches, demonstrating consistent size distributions. The non-pulsed sample shows a larger mean hydrodynamic diameter (∼125 nm), indicating comparatively higher effective particle size. As anticipated, the commercial h-BN powder exhibits the largest characteristic size in DLS, consistent with its micron-scale morphology observed in SEM.

**Figure 3:**
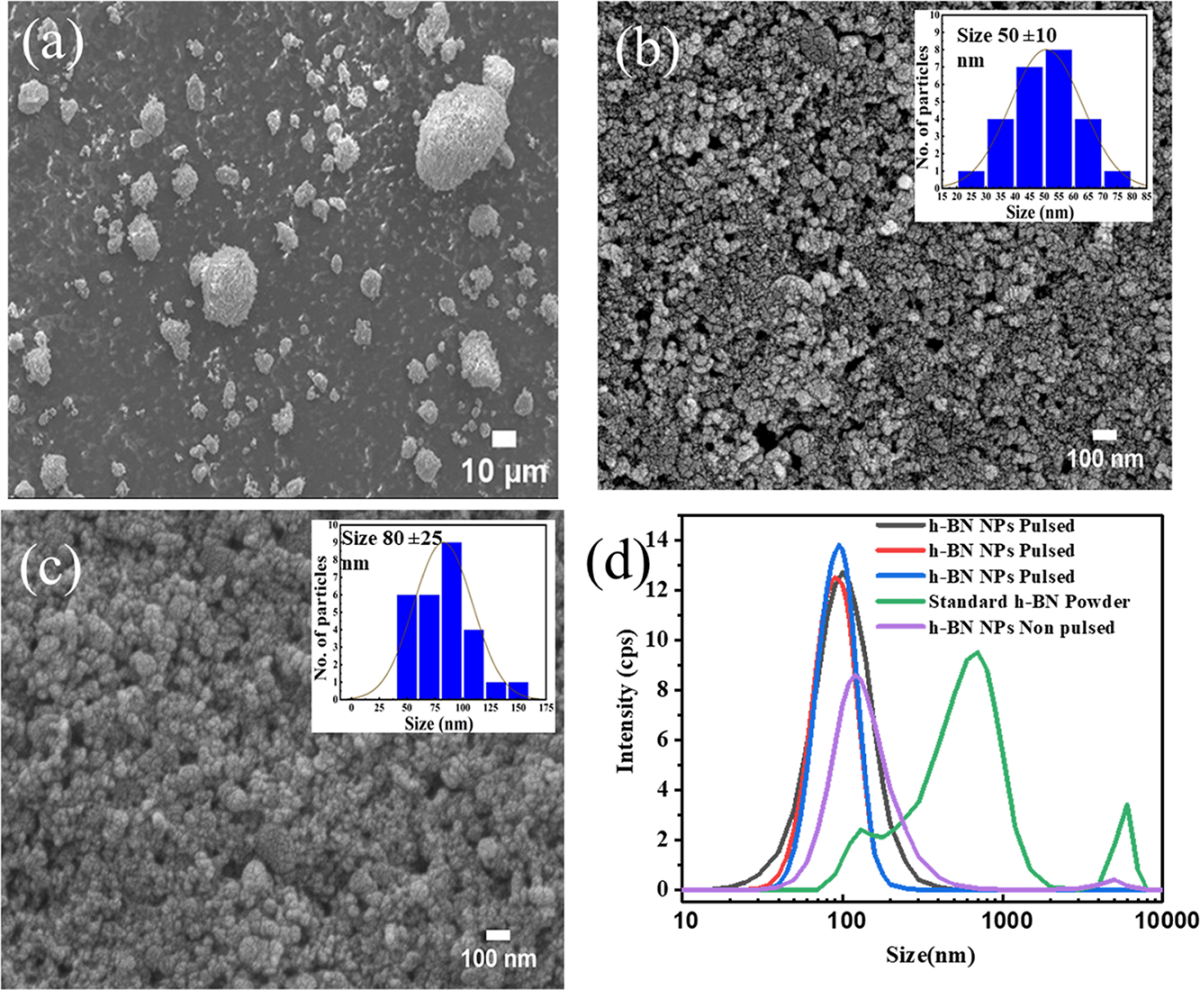
(a) SEM of commercial h-BN powder. (b) Size distribution of pulsed h-BN NPs with ImageJ size distribution inset. (c) SEM of non-pulsed h-BN NPs with ImageJ size distribution inset. (d) DLS size distributions comparing commercial, non-pulsed, and pulsed h-BN samples.

Figure 4 shows the high-resolution transmission electron microscopy (HRTEM) analysis of the pulsed and non-pulsed h-BN samples. Figure 4a represents the pulsed h-BN nanoparticles, which display a significantly smaller particle size distribution, with an average diameter of ∼30 nm. On the other hand, Figure 4b represents the non-pulsed h-BN sample, which displays particles that are significantly larger with an average diameter of ∼75 nm (inset histogram), thus indicating that the pulsed ultrasonication method is significantly more effective in exfoliating the nanoscale particles from the initial material. High-resolution imaging also verifies the retained crystalline structure of the processed h-BN. As seen in Figure 4c, the lattice fringes are resolved in the pulsed sample, and a measured interplanar distance of ∼0.327 nm is in good agreement with the expected basal-plane distance of hexagonal h-BN ((0002) planes). The circled areas in Figure 4c also point out microstructural evolution features related to the pulsing treatment. Area I corresponds to tearing/fragmentation of stacked layers, which is consistent with a mechanical delamination/cleavage mechanism in pulsed ultrasonication, while Area II corresponds to distortion features that can be related to defect creation, e.g., edge dislocation-type contrast, due to high-energy exfoliation and fracture. Figure 4d is a higher-magnification HRTEM image of the pulsed h-BN nanoparticles in which many lattice defects are observed, characteristic of the nanoscale domains formed during size reduction. The corresponding Fast Fourier Transform (FFT) image is shown in the inset of Figure 4d. It displays hexagonal symmetry, further confirming the hexagonal h-BN structure. Figure 4e is the non-pulsed sample with a relatively higher density of lattice defects and disorder, whereas Figure 4f (pulsed sample) displays relatively more continuous lattice fringes with fewer lattice defect features compared to Figure 4e, although with some localized defects.

**Figure 4:**
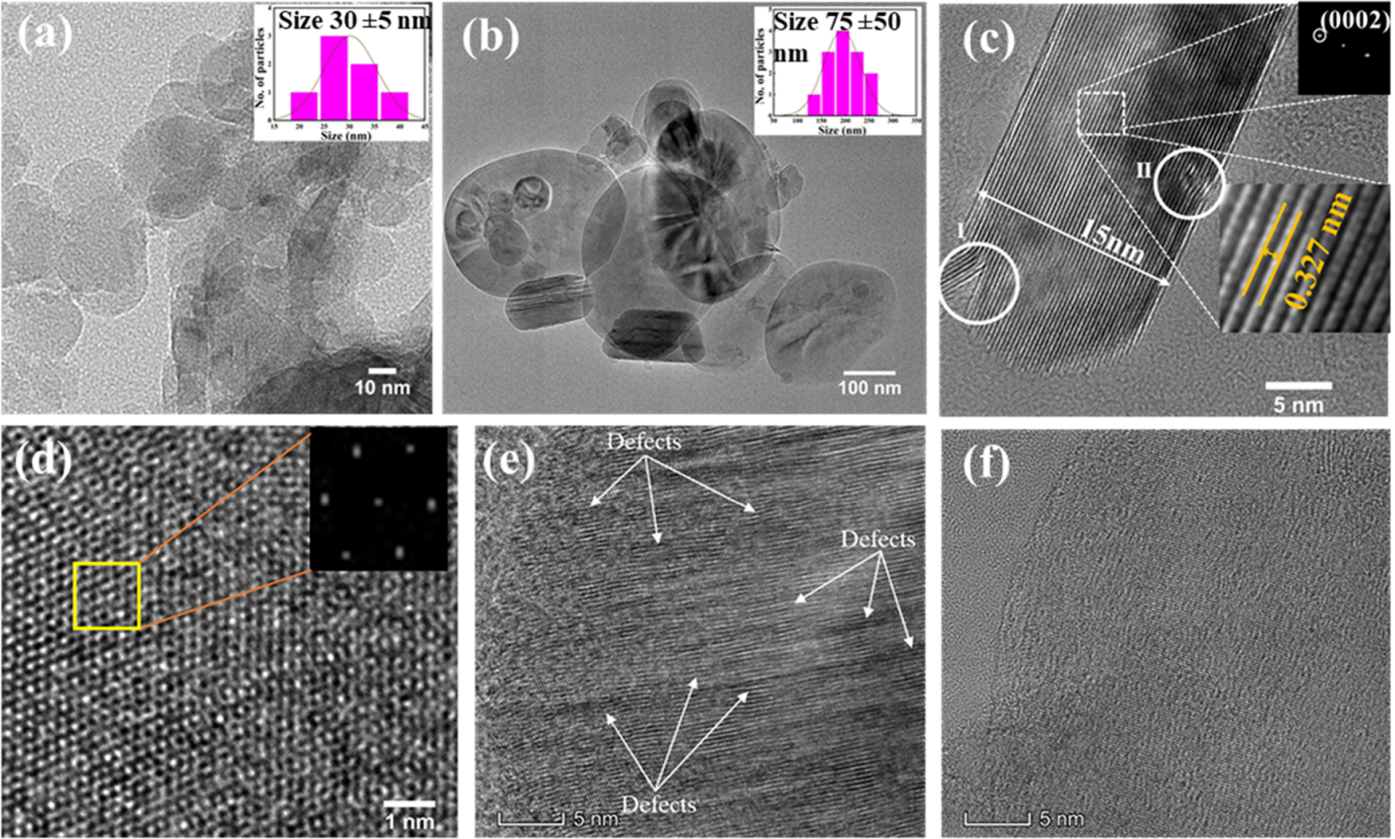
(a) TEM image and size distribution of pulsed h-BN nanoparticles (mean ∼40 nm). (b) TEM image and size distribution of non-pulsed h-BN (mean ∼100 nm). (c) HRTEM of pulsed h-BN showing lattice fringes with *d* ≈ 0.327 nm and representative regions indicating layer fragmentation/defect features; inset diffraction pattern/FFT consistent with h-BN. (d) HRTEM of pulsed h-BN with FFT showing hexagonal symmetry. (e) HRTEM of non-pulsed h-BN highlighting defect-rich regions. (f) HRTEM of pulsed h-BN showing comparatively improved lattice continuity with residual defects.

Since we have coated h-BN NPs and standard h-BN particles on different devices, we use the ratio *R*_OFF_/*R*_ON_ (enhancement ratio) to compare the performance enhancements of these optoelectronic devices. The *R*_OFF_ is the resistance value of the LDR when the UV light is turned OFF, and *R*_ON_ is the resistance value of the LDR when the UV light is turned ON. The *R*_OFF_/*R*_ON_ ratio of all the devices is normalized with respect to the standard LDR *R*_OFF_/*R*_ON_ ratio, such that after normalization, the standard LDR has a maximum *R*_OFF_/*R*_ON_ ratio of 1, so that we can compare the performance of different coated devices. This ratio provides an indication of how much the device’s *R*_OFF_ and *R*_ON_ increased or decreased after coating compared to the commercial LDR. The UV on/off resistance switching and the enhancement ratio for various coating conditions are shown in Figure 5. In Figure 5a, the contrast is made among the commercial (uncoated) LDR, standard h-BN-coated LDR, pulsed h-BN NP-coated LDR, and non-pulsed h-BN-coated LDR. Among the four, the pulsed h-BN NP-coated LDR has the highest difference between the light-OFF and light-ON resistance values, hence having the highest enhancement ratio of 1.35. The standard h-BN-coated LDR, on the other hand, has a relatively lower resistance modulation, hence a lower enhancement ratio of 0.83. The non-pulsed h-BN NP-coated device has a lower enhancement ratio than the pulsed h-BN NP-coated device (0.66). It is observed that the pulsed h-BN NPs-coated LDR exhibits consistently higher peak resistance values compared to the uncoated LDR samples. This suggests that the coating enhances the sensor’s ultraviolet sensitivity. The presence of h-BN improves the light–matter interaction, increasing the sensor’s ability to absorb more UV light, resulting in enhanced photoresponse. A higher resistance value in darkness is due to increased carrier scattering by h-BN NPs and due to deep trap states.^33,34^ Figure 5b shows an enlarged view of a single cycle, clearly indicating that the OFF resistance significantly increases (35%) with the application of the h-BN NPs coating. There is also a significant decrease in ON resistance (23%) of the LDR coated with pulsed h-BN NPs. Hence, the coating almost doubles the sensitivity of the device.

**Figure 5:**
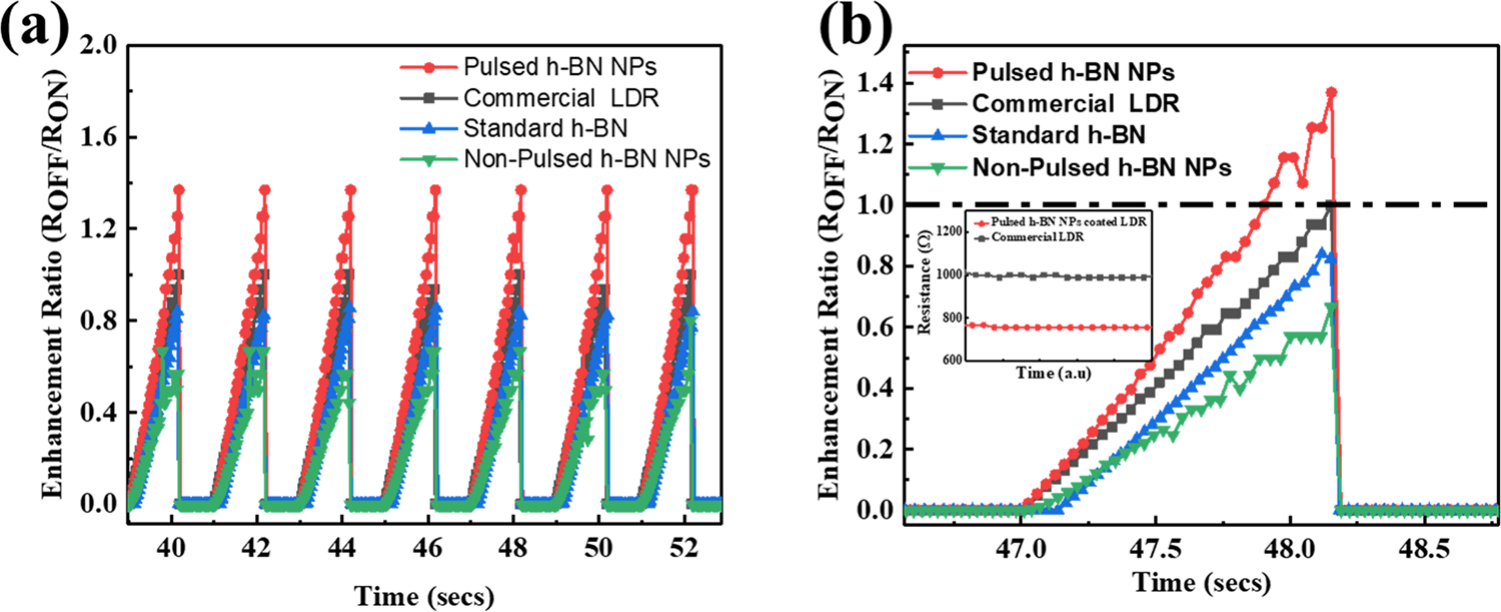
(a) Normalized UV enhancement ratio vs time graph for the commercial (uncoated) LDR, standard h-BN-coated LDR, pulsed h-BN NP-coated LDR, and non-pulsed h-BN NPs-coated LDR under 365 nm illumination for multiple cycles. (b) UV enhancement ratio vs time graph for a single cycle; the inset image contains a zoomed-out section of the graph when the UV light is turned ON.

Device-to-device reproducibility was tested by fabricating and measuring approximately 10 independent devices for each experimental condition. The devices were coated with standard h-BN powder, pulsed h-BN nanoparticles, and non-pulsed h-BN nanoparticles. For each individual device, we measured the enhancement ratio under the same 365 nm illumination and under the same cycling protocol used in every test. The results are given as the average value with one standard deviation and supported by the detailed distribution quantities (median and interquartile range) as shown in Figure 6a. These results illustrate that the pulsed h-BN NPs coating gives the most reproducible enhancement in *R*_OFF_/*R*_ON_ across devices, while the non-pulsed h-BN NPs coating presents a comparably small enhancement ratio. The spread of the data in some of the data sets is mainly due to the device-to-device variability introduced by the solution-processed nanoparticle coatings, rather than experimental uncertainty. It is also important to note that despite the spread of the data, the average enhancement values and relative performance are consistent and repeatable across multiple independently prepared devices. The size of the enhancement is substantially larger than the standard deviation of the data, indicating that the spread of the data is not due to experimental noise. Operational stability and repeatability were checked with an extended UV on/off cycling protocol combined with a time-lapse repeat measurement (Figure 6b). The device was tested initially and retested 2 months later. In this run, the device went through about 400 UV on/off switching cycles under 365 nm wavelength illumination at a fixed bias. As can be seen from Figure 6b, the resistance modulation shows consistency throughout the cycling, while no meaningful drift has been recorded for *R*_OFF_ and *R*_ON_. This shows only negligible degradation during a prolonged operation. All photoresponse characteristics, such as *R*_OFF_/*R*_ON_ and the overall change in resistance, remained essentially the same compared to initial measurements. These continuities confirm that the device preserves its sensing performance over time and supports operational stability and repeatability.

**Figure 6:**
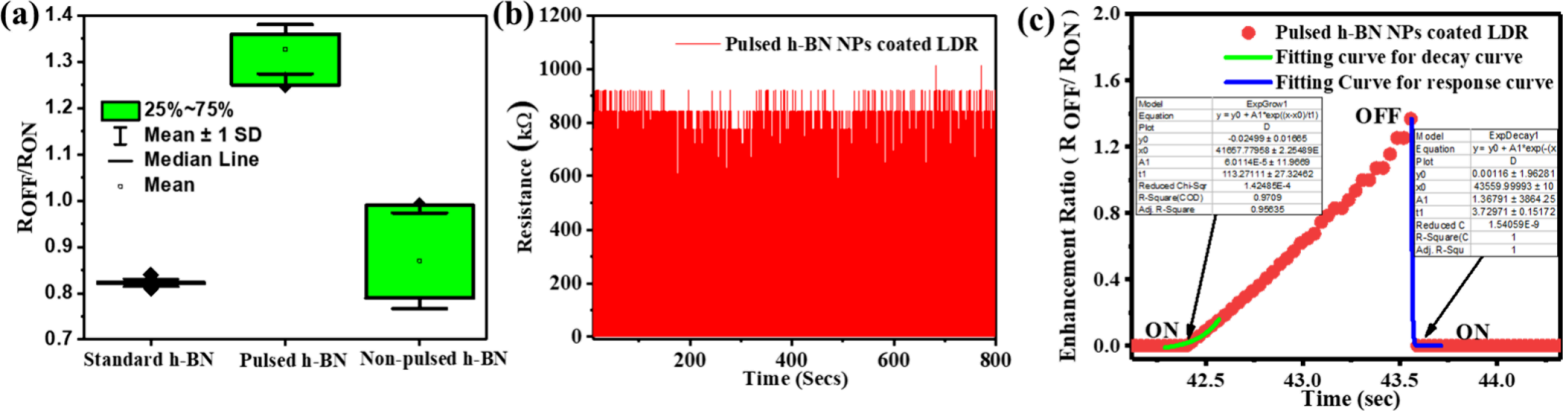
(a) Device-to-device reproducibility of the enhancement ratio (*R*_OFF_/*R*_ON_) for standard, pulsed, and non-pulsed h-BN coatings (mean ± SD with distribution). (b) Long-term operational stability of the device under extended 365 nm UV on/off cycling (∼400 cycles). (c) Curve fitting is used to extract the response and decay times.

The response time (τ_r_) and decay time (τ_d_) of the device before and after the coating were obtained by using the equation *R* = *R*_0_ + *A*e^(±(*x* – *x*_0_)/τ_r_)^ and *R* = *R*_0_ + *A*e^(±(*x* – *x*_0_)/τ_d_)^ (Figure 6c). Based on the equation, the response time and decay time of the h-BN NP-coated sample were calculated; the results are presented in Table 1. There is not much change in response and decay time for the pulsed h-BN NP-coated device compared to the standard uncoated device. Hence, the coating helps to improve the enhancement ratio of the device without affecting the quick decay and response time of the device.

**Table 1:** Decay and Response Time of the Devices Fabricated along with the Fitting Parameter

**Devices**	**Enhancement Ratio**	**Decay time (ms)**	**Response time (ms)**	**Standard deviation**	**Maximum**	**Median**	**Minimum**
commercial LDR	1.00	96 ± 27.0	2.98 ± 0.02	NA	NA	NA	NA
standard h-BN powder-coated LDR	0.83	113 ± 27.0	3.47 ± 0.15	0.0088	0.84	0.824	0.81
pulsed h-BN NP-coated LDR	1.35	121.9 ± 29.0	3.72 ± 0.08	0.05314	1.36	1.36	1.25
non-pulsed h-BN NP-coated LDR	0.63	107 ± 6.0	3.81 ± 0.08	0.10328	0.79	0.79	0.99

From Table 2, it is clear that h-BN NPs produced by the pulsed ultrasonication route provides a fast, scalable, and low-temperature way to obtain h-BN nanoparticles and use them as a surface-modifying/coating layer to enhance UV photoresponse. This method can be integrated with existing photodetector architectures (including other resistive or hybrid sensors) as an add-on layer to potentially improve UV sensitivity and resistance/current modulation without requiring complex device fabrication. It should be noted that it is not easy to make a direct comparison of the response and decay times between the different reports. This is because the values are highly dependent on the form of the material, whether it is in the form of nanoparticles, nanosheets, or thin films. In most of the previous reports, h-BN was the primary active layer. In this report, however, h-BN nanoparticles are used as a surface-modifying layer that affects the charge transport in the CdS device. Therefore, Table 2 is intended to provide a relative comparison of the performance.

**Table 2:** Comparison of This Work with UV Photodetectors Reported Earlier

Material	Synthesized method	Response time (ms)	Decay time (ms)	reference
2D boron nitride nanosheets	PLPD	1600	17000	^35^
h-BN nanosheets	solid state-assisted synthesized h-BN nanosheets	200	500	^22^
GaN nanostructures	plasma-assisted MBE	18	27	^36^
nonpolar GaN	PAMBE	280	450	^37^
Pt/ZnO	flame spray pyrolysis (FSP) deposition	9600	74000	^38^
Se/ZnO	CVD	0.69	13.5	^39^
h-BN NP coated	ultrasonication exfoliation	3.59	121	this work

Figure 7 summarizes the X-ray photoelectron spectroscopy (XPS) investigation of h-BN synthesized using pulsed, non-pulsed, and pure h-BN conditions, providing comprehensive insight into the surface composition, chemical bonding, and defect chemistry. The survey spectra of the pulsed (Figure 7a), non-pulsed (Figure 7d), and pure (Figure 7g) samples are dominated by the characteristic B 1s and N 1s photoemission peaks, confirming the successful formation of boron nitride in all cases. No metallic impurities or extraneous elements are detected within the sensitivity limits of XPS, indicating a high chemical purity. A weak C 1s signal is observed in all samples and is attributed to adventitious carbon adsorption arising from air exposure during sample transfer, which is commonly reported for BN-based materials.

**Figure 7:**
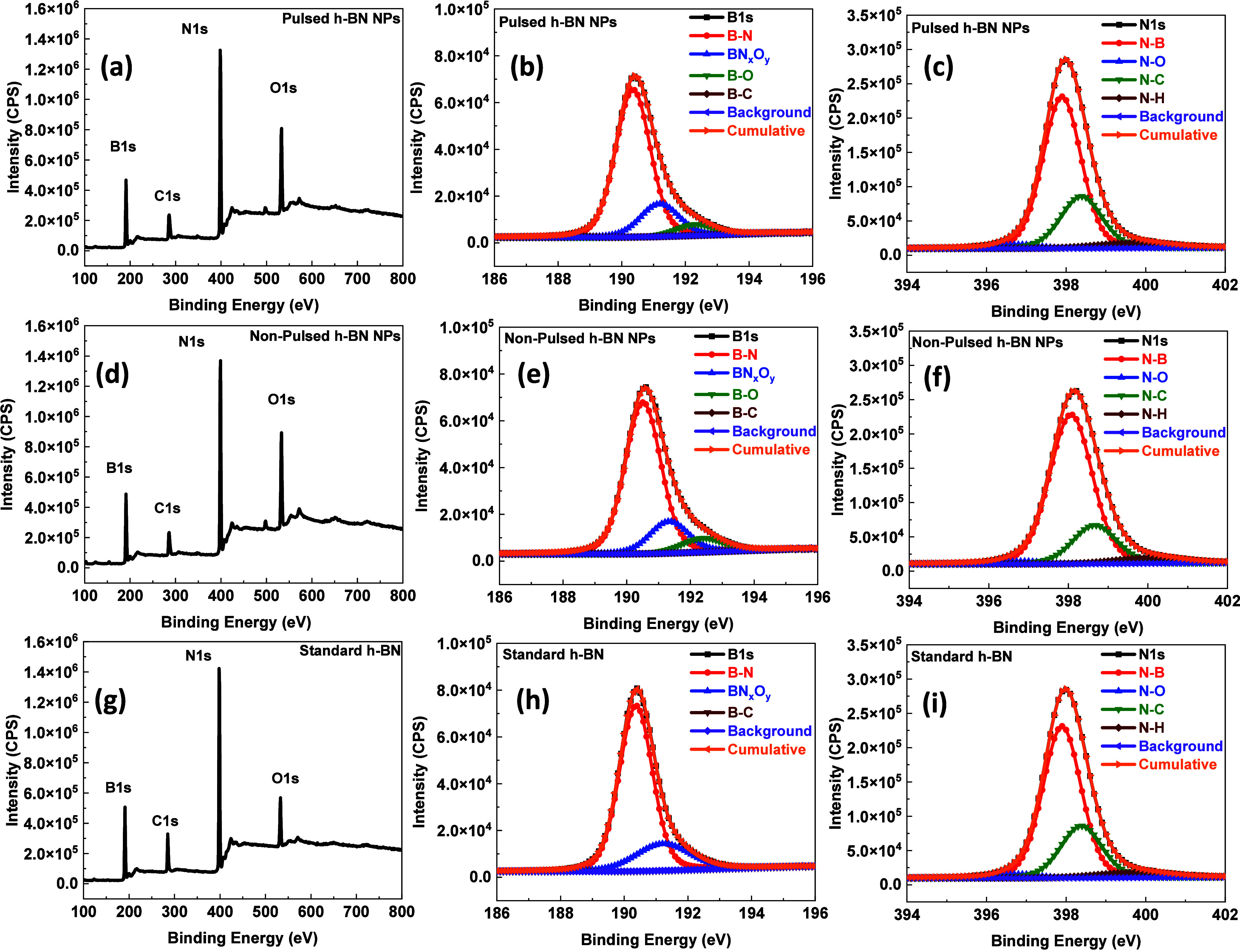
High-resolution XPS spectra of h-BN synthesized via pulsed, non-pulsed, and pure routes: (a–c) B 1s and N 1s spectra of pulsed h-BN, (d–f) B 1s and N 1s spectra of non-pulsed h-BN, and (g–i) B 1s and N 1s spectra of pure h-BN. Deconvoluted components corresponding to B–N, B–O, BN_*x*_O_*y*_, B–C, N–B, N–O, N–C, and N–H species are shown along with fitted envelopes and backgrounds, highlighting synthesis-dependent defect chemistry and oxidation states.

To gain deeper insight into the bonding configurations and electronic states, high-resolution XPS spectra were acquired for the B 1s, N 1s, O 1s, and C 1s regions. All binding energies were calibrated with respect to the adventitious carbon C 1s peak at 284.6 eV to ensure accurate energy referencing. Spectral fitting was performed using a U2 Tougaard background subtraction combined with a Lorentzian asymmetric line shape function, [LA(50)], enabling reliable deconvolution of overlapping spectral features, lattice, and defect-related components. High-resolution analysis of the B 1s region reveals clear differences in boron-bonding environments induced by the growth mode. For the pulsed h-BN sample (Figure 7b), the dominant B 1s peak centered at 190.35 eV is assigned to sp^2^-hybridized B–N bonding, confirming the formation of hexagonal boron nitride, while a minor component at ∼188.8 eV corresponds to B–C bonding, indicating a small contribution from carbon-related surface species.^40,41^ Additional components at 191.19 and 192.30 eV are assigned to mixed BN_*x*_O_*y*_ ^42,43^ and B–O^44^ bonding configurations, respectively, indicating partial oxygen incorporation.^45^ Importantly, the B–O contribution in the pulsed sample is relatively weak (area ≈ 7668) and the B–N peak exhibits a narrow full width half maximum (FWHM ≈ 1.28 eV), reflecting improved bonding uniformity and reduced boron-site disorder. These features suggest that pulsed growth suppresses severe lattice disruption, while allowing limited surface oxidation. The N 1s spectra further elucidate the role of pulsed synthesis in defect redistribution. In the pulsed sample (Figure 7C), the N 1s region is dominated by the N–B lattice peak at 397.9 eV, accompanied by discernible contributions from N–O (396.89 eV), N–C (398.44 eV), and N–H (399.71 eV) species.^46,47^ The peak position, FWHM, areas, and normalized defect ratio of the prepared samples are shown in Table 3. The relatively high N–O peak area (≈ 9081) indicates that oxygen incorporation in the pulsed film occurs preferentially at nitrogen sites rather than forming B–O configurations at boron sites. This nitrogen-centered oxidation pathway is further supported by the reduced B–O intensity observed in the B 1s spectrum. The presence of N–C and N–H species reflects surface termination and adsorption effects, which are expected for high-surface-area BN materials and do not necessarily indicate bulk lattice degradation.

**Table 3:** Deconvoluted B 1s and N 1s XPS Peak Positions, FWHM, Areas, and Normalized Defect Ratios for Pulsed, Non-pulsed, and Pure h-BN, Highlighting Differences in Oxidation and Vacancy-Related Defect Densities

		Pulsed			Non- Pulsed			Pure		
Region	Name	Position	FWHM	Area	Position	FWHM	Area	Position	FWHM	Area
B 1s	B–C	188.810	0.740	370.620	188.840	1.000	312.620	188.830	0.720	494.330
	B–N	190.350	1.280	92423.310	190.510	1.310	97862.450	190.370	1.220	99082.180
	BN_*x*_O*_y_*	191.190	1.420	22774.840	191.330	1.420	22238.980	191.20	2.000	26353.970
	B–O	192.300	1.400	7668.880	192.380	1.570	10755.440			
N 1s	N–O	396.890	1.450	9080.980	396.830	1.170	5210.980	396.830	1.120	7177.410
	N–B	397.900	1.200	279567.530	398.070	1.250	313181.040	397.900	1.120	285193.310
	N–C	398.440	1.260	90774.980	398.660	1.300	83958.540	398.380	1.230	106763.540
	N–H	399.710	2.080	23949.130	400.000	1.970	17777.450	399.640	2.120	17598.030
	BN_*x*_O*_y_*/B–N	0.246			0.227			0.226		
	B–O/B–N	0.083			0.110					
	N–O/N–B	0.032			0.017			0.025		
	N–C/N–B	0.325			0.268			0.374		
	N–H/N–B	0.086			0.057			0.062		

In contrast, the non-pulsed h-BN sample (Figure 7e) shows a more pronounced degree of boron oxidation. While the primary B–N peak at 190.51 eV remains dominant, the relative intensities of the BN_*x*_O_*y*_ (191.33 eV) and B–O (192.38 eV) components are significantly higher than those in the pulsed sample. The B–O peak area reaches approximately 10,755, indicating enhanced formation of oxygen coordination environments around boron atoms. This observation implies that, in the absence of pulsed processing, oxygen incorporation preferentially disrupts the BN lattice, leading to a higher density of boron-centered defects. The standard h-BN reference (Figure 7h) exhibits the sharpest B 1s spectrum, dominated by a strong B–N peak at 190.37 eV with the largest integrated area (≈99,082) and no detectable B–O component, confirming superior lattice integrity and minimal oxygen-induced disorder. Further in Figure 7f, the N 1s spectrum depicts that dominated by the N–B peak at 398.07 eV with comparatively weaker N–O contributions (area ≈5211), suggesting that nitrogen oxidation is less pronounced than in the pulsed film (Table 3). This asymmetry between boron and nitrogen oxidation confirms that, under non-pulsed conditions, oxygen preferentially bonds to boron, leading to extensive B–O formation and greater lattice distortion. The pure h-BN sample (Figure 7i) shows a dominant and sharp N–B peak at 397.9 eV, with minor N–O, N–C, and N–H components attributed mainly to surface termination rather than intrinsic defects, consistent with its higher chemical uniformity.

Overall, the comparative XPS analysis in Figure 7 demonstrates that pulsed synthesis fundamentally alters the oxidation pathway and defect distribution in h-BN. Boron-related defects, primarily associated with oxidation-induced boron vacancies, were assessed by using the BN_*x*_O_*y*_/B–N and B–O/B–N ratios. The pulsed h-BN sample shows a comparable BN_*x*_O_*y*_/B–N ratio of 0.246 but a markedly reduced B–O/B–N ratio of 0.083, demonstrating that pulsed synthesis effectively suppresses the formation of mixed BN_*x*_O_*y*_ environments while maintaining controlled surface oxidation. This reduction in the BN_*x*_O_*y*_ content reflects a lower density of deep boron-related traps, which is advantageous for dielectric stability and exciton transport. The pure h-BN reference exhibits a −BN_*x*_O_*y*_/B–N ratio of 0.266; however, the absence of a detectable B–O component indicates that oxygen is largely confined to surface termination rather than associated with bulk boron vacancy formation. In contrast, the non-pulsed h-BN sample exhibits a -BN_*x*_O_*y*_/B–N ratio of 0.227 together with a relatively high B–O/B-N ratio of 0.110, indicating extensive oxidation of boron-deficient sites and significant disruption of the BN lattice (Table 3). Such a high density of boron-centered defects is known to introduce deep trap states that act as nonradiative recombination centers and impede carrier transport in optoelectronic devices. Nitrogen-related defects were similarly analyzed using the N–O/N–B, N–C/N–B, and N–H/N–B ratios, which probe nitrogen vacancy formation and associated π–π* loss features. The non-pulsed h-BN sample shows a low N–O/N–B ratio of 0.017 but a substantial N–C/N–B ratio of 0.268, suggesting that nitrogen vacancies are predominantly passivated by hydrogen-containing species, a signature of disordered regions that introduce donor-like states and enhance charge trapping. In comparison, pulsed h-BN exhibits a significantly higher N–O/N–B ratio of 0.033, nearly twice that of the non-pulsed sample, along with an increased N–C/N–B ratio of 0.325. This trend indicates enhanced formation of controlled nitrogen vacancies and edge-like sp^2^ domains, which are commonly associated with pronounced π–π* shakeup loss features and exciton localization in h-BN. The pure h-BN sample displays an intermediate N–O/N–B ratio of 0.025 and the highest N–H/N-B ratio of 0.374, consistent with predominantly surface-passivated nitrogen defects rather than bulk vacancy formation (Table 3). Overall, these quantitative defect metrics demonstrate that pulsed synthesis redistributes defect chemistry by suppressing disruptive boron-centered B–O defects while enabling controlled nitrogen-related states, thereby creating a defect landscape that is more favorable for optoelectronic functionality than that of non-pulsed h-BN NPs.

### Charge Transfer Mechanism

Analysis of XPS data shows that both pulsed and non-pulsed h-BN NPs have a tendency toward oxygen bonding, although the type of bonding is vastly different depending on the synthesis method. The pulsed h-BN has a preference for N–O related bonds, while the non-pulsed h-BN has a higher tendency toward B–O bonds. The energy level created by this oxide formed in the non-pulsed h-BN sample is closer to the conduction band of the h-BN,^48^ and it results in poor band bending and hence results in poor charge transport in non-pulsed h-BN particle-coated devices. In the case of pulsed h-BN NPs, we could see that the oxygen nitrogen bond is large in quantity (XPS data), and this shifts the Fermi level closer to the valence band region. This results in a smoother band bend and better charge flow between h-BN and CdS. In our case, we therefore assume that in the pulsed sample, there is a downward shift in the Fermi energy level and this results in better band alignment and charge transfer, and in the case of non-pulsed sample, there is an upward shift in Fermi energy level,^49^ which results in poor band bending that hinders flow of electrons. It is important to point out that the present CdS­(LDR)/h-BN nanoparticle heterostructure is a nonideal solution-processed heterostructure, where the interface is defined by nanoparticle dispersion, surface defects, and mixed bonding configurations rather than atomically abrupt interfaces. Therefore, the charge-transfer and trap-assisted photoconduction model described in this article is a model that describes the dominant experimental trends rather than a rigorous description of the interfacial band alignment. A more accurate determination of the work function and Fermi-level position for the pulsed and non-pulsed h-BN samples would require ultraviolet photoelectron spectroscopy (UPS); such UPS measurements are beyond the scope of the present work and will be pursued in future studies. The band alignment and charge transfer is shown in Figure 8a,b (values for h-BN and CdS are taken from refs 50 and 9).

**Figure 8:**
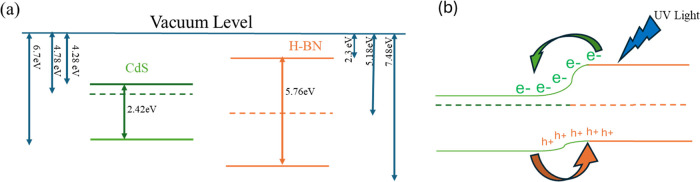
(a) Schematic band alignment of CdS (LDR) and h-BN before contact. (b) After contact, Fermi-level equilibration induces interfacial band bending and UV-driven charge transfer across the CdS/h-BN interface.

## Conclusions

The present experiment successfully created a device with enhanced UV light-sensing properties. The h-BN NPs having a size of ∼30 nm were synthesized by the pulsed ultrasonification method for this purpose. The synthesized particles are uniformly distributed on the surface of the LDRs to provide better coverage for UV light sensing. The combination of uncoated, standard h-BN, non-pulsed h-BN, and pulsed h-BN nanoparticles coated devices provides a controlled framework to isolate the effect of synthesis-induced structural modification on device performance. In insulating materials, edge dislocations and screw dislocations create a chain of broken bonds, which act as deep traps that could capture electrons and holes.^51^

The photoresponse in a nanostructured modified photoconductor is often dominated by trap-assisted photoconduction, where defect/surface states control carrier lifetime and the effective conductivity of the transport channel. The photogenerated charge separates at the interface in nanoparticles and one carrier gets trapped for a long amount of time while the other countercarrier circulates repeatedly through the high-mobility channel, yielding large gain (conceptually *G* ∼ τ_life_/τ_transit_). This leads to a decrease in *R*_ON_ resistance of the device.^52^ Photoexcitation → charge separation → trapping of one carrier species → sustained modulation of the transport pathway has also been proposed directly in h-BN photoconductive detectors. Zheng et al. show that edges/steps in few-layer h-BN serve as trap sites and that under illumination, a portion of the photoexcited carriers is trapped at these sites, resulting in persistent photoconductivity and increased responsivity.^53^ When the UV light source is switched off, the replenishment of the photogenerated carriers ceases, and the trapped charges start to detrap (or release) from the defect/interface states. The released carriers recombine with the remaining free carriers, which decreases the free carrier concentration in the transport channel, thus reversing the device to the dark state. Consequently, the resistance increases very rapidly to higher values in pulsed h-BN.

In the current CdS­(LDR)/h-BN NP device, we hypothesize that the h-BN NPs can provide additional defect/surface states and interface states that can trap the photoexcited carriers under UV illumination. The trapped charge can provide a local electrostatic gate effect (photogating-like mechanism) and/or prolong the effective carrier lifetime in the CdS, leading to an enhancement of the resistance modulation (larger *R*_OFF_/*R*_ON_ values). Further work (e.g., direct work-function/trap spectroscopy or UPS/KPFM) would be required to quantitatively determine the trap energetics and their exact contribution in the present CdS­(LDR)/h-BN NP system. Non-pulsed h-BN NPs have more defect states when compared with pulsed h-BN NPs and thus give a lower enhancement ratio. Although moderate trap density can be beneficial for UV detection by allowing carrier trapping and detrapping and hence enhancing the photoresponse, a high trap density is not ideal. This is because a high trap density results in increased scattering^54^ and nonradiative recombination rates, as well as an increased localized state density that hinders efficient carrier transport.^55^ The nonmonotonic behavior of the relationship between performance and defect density indicates the coexistence of competing factors: an optimal level of trap density can improve photogating, while a high defect density can increase recombination and scattering.

Although defect states in h-BN may enable sub-bandgap absorption, h-BN possesses an ultrawide bandgap (∼5.8–6.0 eV), while the excitation wavelength used in this study (365 nm, ∼3.4 eV) is significantly lower in energy. Consequently, any photoconductivity originating from h-BN is expected to be weak and defect-assisted. Furthermore, the deposited h-BN nanoparticles form a thin and noncontinuous layer that does not provide a percolated conduction pathway between the electrodes; therefore, photogenerated carriers within h-BN are unlikely to directly contribute to the measured current and are more likely to undergo recombination or trapping.^56^ The nearly unchanged response and decay times after h-BN coating further support the notion that the measured photocurrent predominantly arises from the underlying device.

We point out that further control experiments, such as thickness-controlled coatings, inert nanoparticle layers, or different device substrates, could further test or refine this interpretation by isolating individual contributing factors. The sensitivity of the device increases with the coating of the h-BN NPs. This technique can be incorporated into the design of conventional photodetectors (resistive or hybrid) as a simple addition at the interface that could possibly improve the UV sensitivity and resistance/current modulation. Incorporating these faces challenges such as achieving uniform deposition of h-BN nanoparticles and mitigating the influence of humidity and surface adsorbates on device performance. Addressing these limitations represents an important scope for future studies toward integrating h-BN nanoparticles into conventional photodetector platforms. Therefore, the coating with h-BN NPs proves to be a facile method for enhancing the UV photoresponse, thereby leading to improved quantum and semiconductor sensors.
